# Long-Term Impact of Radiation on the Stem Cell and Oligodendrocyte Precursors in the Brain

**DOI:** 10.1371/journal.pone.0000588

**Published:** 2007-07-11

**Authors:** Georgia Panagiotakos, George Alshamy, Bill Chan, Rory Abrams, Edward Greenberg, Amit Saxena, Michelle Bradbury, Mark Edgar, Philip Gutin, Viviane Tabar

**Affiliations:** 1 Department of Neurosurgery, Sloan-Kettering Institute for Cancer Research, New York, New York, United States of America; 2 Department of Radiology, Sloan-Kettering Institute for Cancer Research, New York, New York, United States of America; 3 Department of Pathology, Sloan-Kettering Institute for Cancer Research, New York, New York, United States of America; University of Massachusetts, United States of America

## Abstract

**Background:**

The cellular basis of long term radiation damage in the brain is not fully understood.

**Methods and Findings:**

We administered a dose of 25Gy to adult rat brains while shielding the olfactory bulbs. Quantitative analyses were serially performed on different brain regions over 15 months. Our data reveal an immediate and permanent suppression of SVZ proliferation and neurogenesis. The olfactory bulb demonstrates a transient but remarkable SVZ-independent ability for compensation and maintenance of the calretinin interneuron population. The oligodendrocyte compartment exhibits a complex pattern of limited proliferation of NG2 progenitors but steady loss of the oligodendroglial antigen O4. As of nine months post radiation, diffuse demyelination starts in all irradiated brains. Counts of capillary segments and length demonstrate significant loss one day post radiation but swift and persistent recovery of the vasculature up to 15 months post XRT. MRI imaging confirms loss of volume of the corpus callosum and early signs of demyelination at 12 months. Ultrastructural analysis demonstrates progressive degradation of myelin sheaths with axonal preservation. Areas of focal necrosis appear beyond 15 months and are preceded by widespread demyelination. Human white matter specimens obtained post-radiation confirm early loss of oligodendrocyte progenitors and delayed onset of myelin sheath fragmentation with preserved capillaries.

**Conclusions:**

This study demonstrates that long term radiation injury is associated with irreversible damage to the neural stem cell compartment in the rodent SVZ and loss of oligodendrocyte precursor cells in both rodent and human brain. Delayed onset demyelination precedes focal necrosis and is likely due to the loss of oligodendrocyte precursors and the inability of the stem cell compartment to compensate for this loss.

## Introduction

Radiation therapy is a powerful tool in the treatment of primary and metastatic cancers of the brain. However, tissue tolerance of the normal brain is very limited and radiation doses have to be tailored to minimize the deleterious effect on the nervous system [1]. The late effects of radiation are of particular clinical relevance and are manifest as cognitive impairment. There is currently no effective treatment for radiation-induced cognitive decline[2], [3]. While the pathogenesis is not fully understood, studies of brain irradiation in humans[4] and animals[5] suggest the loss of myelin sheaths with apparent preservation of axons. Vascular changes, such as thrombosis and hyalinization are also seen, particularly at high doses and in the subacute phase[6]. There are controversial views as to the relative importance of the vascular theory versus the glial theory as a prime underlying element of pathogenesis of late radiation effects [7]. Histological studies of irradiated brains essentially predate our current understanding of precursor biology in the adult CNS. It is now recognized that there are two major specialized zones of cell proliferation in the adult brain: the subventricular zone (SVZ) and the dentate gyrus. These regions contain stem cell and precursor populations that self-renew and generate neurons and glia throughout life[8], [9]. Outside these regions the majority of the cycling cells in the adult brain (>75%) are oligodendroglial progenitors, identified by their expression of NG2 proteoglycan, PDGFRA or O4[10], [11].

It was recently shown that irradiation leads to a dose-dependent loss of cell types in the subventricular zone (SVZ) with impairment of SVZ repopulation up to three months[12]. Older studies have also reported a decrease in mitotic activity non-specifically in the “subependymal plate” after different radiation doses with subsequent delayed recovery[13]. Additionally, there is loss of granule cells in the hippocampus up to 3 months after brain irradiation [14]. The effects of brain irradiation on oligodendrocyte progenitors is described in the spinal cord whereby exposure of short segments to high dose radiation (40 Gy) results in a decrease in the number of NG2+cells by nearly 50%[15] [16] but this data was not extended over long time periods or to the brain itself. It is unclear from current literature if the loss of oligodendrocyte progenitors is permanent or if delayed recovery occurs. The loss of these cells may underlie the absence of remyelination in the late phases of radiation. Conversely radiation may result in alterations of the microenvironment that inhibit survival of newly born oligodendroglial progenitors and/or their maturation into the myelin phenotype.

In this study, we quantitate the impact of whole brain irradiation on the SVZ compartment and olfactory neurogenesis, as well as on the oligodendrocyte progenitors and mature myelin. Animals are followed over a period of 15 months thus allowing a detailed understanding of the kinetics of the stem cell and progenitor subpopulations both in normal aging and post radiation. For additional validation of these findings we analyzed human tissues post radiation. Such tissues are rarely available for analysis and were obtained from surgical specimens collected over six years. Data from rat and human tissues that had been irradiated at various periods prior to analysis suggest a similar pattern of oligodendroglial progenitor loss and demyelination over time post radiation. There was no obvious vascular damage despite ultrastructural evidence of myelin fragmentation. To our knowledge this is the first long term study of the impact of high dose therapeutic range irradiation on specific cell subpopulations in the subventricular zone and oligodendrocyte progeny in the brain.

## Methods

### X-irradiation

Young adult Sprague Dawley female rats (Taconic; 3 to 15 months old) were used throughout the study. A dose of 25 Gray (Gy) was administered to the cranium of 3-month-old rats using a 250kV-orthovoltage system equipped with a 0.25mm copper filter. A custom-designed positioning device platform based on the standard stereotactic frame was used so that six animals could be simultaneously irradiated. Animals were fully anesthetized using a combination of Ketamine (90 mg/kg ip) and Xylazine (4 mg/kg ip) prior to being placed in the frame. The heads were centered in a 20 cm×20 cm treatment field and x-irradiation was limited to an adjustable 2 cm circular aperture centered over the cranium. A lead plate shielded the rest of the body, including the animals' ears, hindbrain, and orbits; the olfactory bulb was spared. The beam was directed onto the head at a source to skin distance of 21 cm at a calculated angle of 5.7° from vertical. An X-ray of the animals in their final position was taken and developed in double exposure (with and without lead shielding) to check the appropriate skull position against an X-ray of the “ideal” position previously confirmed by dose calibration tests. The full radiation dose is administered after final adjustment. Dosimetry was performed by implanting lithium fluoride thermoluminescent dosimeters into various areas of the brain as well as protected regions (ears, oropharynx, orbit and hindbrain). The corrected dose rate was determined to be 117.5cGy/min with a calculated dose variation at a maximum of 9% per 5 mm from the center of the field in the dorsoventral axis. Instrument calibration was performed regularly by the department of medical physics. Rats were irradiated in batches of 6 animals per set; several sets were done per week and the animals distributed randomly into time point groups at n = 4 per time point.

### Bromodeoxyuridine (BrdU) Administration

For three days prior to sacrifice, irradiated and control rats were injected daily with 300 mg/kg BrdU (97%, Aldrich) in sterile normal saline intraperitoneally.

### Tissue Processing

Briefly, rats were deeply anesthetized with an intraperitoneal injection of an overdose of Pentobarbital Sodium (Nembutal Sodium Solution; Abbot Laboratories), followed by transcardial perfusion of 0.1% heparinized normal saline (Sigma) at 4°C and an equal volume of 4% paraformaldehyde (PFA) in PBS also at 4°C (pH 7.4). Brains were then carefully extracted, placed in 4% PFA for overnight fixation at 4°C and subsequently transferred to 30% sucrose at 4°C until embedding. Optimal Cutting Temperature Compound (O.C.T. Compound, Tissue Tek) was used for embedding and 25 µm or 10 µm (for histology) sections were cut on a freezing cryostat. Sections were stored at −80°C until use for immunohistochemical analyses.

### Immunohistochemistry

Sections were washed briefly with PBS 0.1% BSA and blocked for fluorescence immunohistochemistry with 10% normal goat serum (NGS, Gibco) in PBS 0.1% BSA and 0.3% Triton X-100 for one hour (Triton X-100 was omitted for surface antigens). Pretreatment steps were performed for some antibodies as follows: 2N HCl for 30 min at 25°C for BrdU and 100% methanol for 7 min at −20°C for MAG. Primary antibodies were incubated overnight at 4°C and appropriate secondary antibodies (Alexa conjugates, Molecular Probes) were applied on the following day at 25°C for one hour. Slides were then washed in PBS, counterstained with the nuclear marker DAPI (Molecular Probes) and mounted in 70% glycerol. The primary antibodies used included: BrdU (1∶50, BD); chondroitin sulfate proteoglycan NG2 (1∶200, Chemicon); guinea pig Doublecortin (DCX, 1∶3000, Chemicon); Calretinin (1∶1000, Swant); Rat Endothelial Cell Antigen (RECA, 1∶100, Serotec); rat Myelin Basic Protein (MBP, 1∶200, Chemicon); Myelin-Associated Glycoprotein (MAG, 1∶100, Chemicon); O4 (1∶100, Chemicon); O1 (1∶100, Chemicon); CNPase (1∶200, Sternberger Monoclonals); PDGFRA (1∶50, Santa Cruz Biotechnology); Neurofilament M (1∶200, Chemicon); Neurofilament 70kDa (1∶200, Chemicon); Galactocerebroside (Galc, 1∶200, Chemicon); von Willebrand Factor (vWF, 1∶100, BD Biosciences Pharmingen).

### Stereological Analyses/Cell Counts

All stereological analyses were conducted by a trained operator with no knowledge of animal identification. Total number of proliferating cells (BrdU+) and proliferating oligodendrocyte progenitor cells (BrdU/NG2+) was assessed separately in the SVZ, cortex and corpus callosum by stereological methods using the optical fractionator probe (Stereo Investigator Version 6, Micro Brightfield). Fractionator probes were designed and applied using the stereological software with the coefficient of error (Gundersen) set at ≤0.04. Systematic random sampling was applied to each of the three regions of interest as defined on serial sections selected at discrete intervals with a random start. Data is presented as average estimated total cell number at each time point and for appropriately age-matched controls.

For stereological analysis of endothelial cells, serial sections of cortex and corpus callosum stained for RECA (1∶100, Serotec) were analyzed for the following: total capillary segment number using the optical fractionator method and total capillary length using the virtual sphere method[17], [18]. Unbiased counting frames with inclusion/exclusion lines were used to avoid edge effects.

For the olfactory bulb, regions of interest were identified as the anterior extension of the rostral migratory stream (distal or rostral RMS), the granular cell layer, the glomerular layer, and the entire olfactory bulb. We counted the total number of proliferating cells (BrdU+) in the olfactory bulb, in addition to the number of proliferating migrating neuroblasts in the anterior RMS (BrdU/DCX+), the number of DCX+neuroblasts in the granular layer, and the number of Calretinin+periglomerular interneurons. All time-points were compared to cell counts from age-matched controls. Statistical testing performed using ANOVA followed by post-hoc analysis (Newman-Keuls). Data is presented as mean cell number±standard error.

### Fluorescence Intensity Quantification

Measurements of fluorescence staining intensity were made on digital images obtained from cryopreserved brain specimens stained for MBP, MAG, and O4 using NIH ImageJ software. Random sections were selected from a pre-defined region of interest that encompassed the corpus callosum from genu to splenium. The sections were immunostained concomitantly using strictly identical immunohistochemistry protocols and the same antibody lots. Intensity was analyzed in two regions of the corpus callosum per section at three sections per brain for two animals per time point. Briefly, measurement involved acquiring color images at the same exposure level, converting images to 8-bit gray scale (fluorescence intensity from 0 to 255), and calculating mean intensity in the region from thresholded pixels excluding background fluorescence. Statistical testing performed using ANOVA followed by post-hoc analysis (Newman-Keuls).Data is presented as mean±standard error.

### Human Tissue

Human normal brain and radiated white matter samples were obtained intraoperatively at various time points following whole brain irradiation. Tissues were obtained after patients' written consent under a general tissue collection protocol approved by the institution's Institutional Review Board (IRB). Specific experimental use of the tissue was also approved by the Human Tissue Utilization Committee (HTUC) and the IRB. Human material consisted of glial tissue in the immediate vicinity of brain lesions. Patients had either received no radiation or had received radiation as a clinically determined treatment modality at various intervals prior to surgery. Only tissues ascertained to be tumor free by our pathologist were used. Two samples were obtained from patients with clinically symptomatic radiation necrosis rather than tumor recurrence and were analyzed separately. “Normal control” consisted of glial tissue around a lesion in a brain that never received radiation. Samples obtained 2–7 months following irradiation were identified as “early post-irradiation” and samples obtained beyond 9 months after irradiation were classified as “late post-irradiation”. Tissue samples were fixed overnight in 4% PFA in PBS at 4°C and subsequently transferred to 30% sucrose at 4°C until embedding in O.C.T. compound and sectioning at 10um on a freezing cryostat. Sample numbers: controls (n = 7); early post XRT (n = 5); late post XRT (n = 6).

### Electron Microscopy

Tissues from irradiated and control rat brains, as well as from human control and irradiated brain specimens, were processed for electron microscopy by fixation in 5% glutaraldehyde and 2% formaldehyde in 0.075M Cacodylate buffer, followed by postfixation in 1% osmium tetroxide and 1.5% potassium ferricyanide for 1 hour. Tissues were subsequently stained for 1 hour in 1.5% uranyl acetate, dehydrated through a graded ethanol series followed by 100% acetone, embedded in epoxy Embed 812 resin (Electron Microscopy Sciences, Hatfield, PA) and polymerized at 60 degrees C overnight. Semithin (1 µm) and ultrathin (60 nm) sections were cut using a Diatome diamond knife (Diatome USA, Hatfield, PA) on a Leica Ultracut S ultramicrotome. Semithin sections were stained in toluidine blue (pH 2.0–2.5) and ultrathin sections were contrasted with lead citrate for electron microscopy. Ultrathin sections were viewed on a JSM 100 CX-11 electron microscope (JEOL USA, Inc., Peabody, MA) and images recorded on Kodak 4489 Electron Image film and subsequently digitized on an Epson Expression 1600 Pro Scanner at 900dpi. These procedures were performed at EM core facilities at Sloan Kettering and Cornell University.

### MRI

Female irradiated (n = 3) and age-matched non-irradiated rats (n = 1) were anesthetized using 1.5–2% isoflurane in a 70%N_2_O+30%O_2_ mixture. *In vivo* magnetic resonance (MR) imaging experiments were performed on a Bruker Biospec 4.7-Tesla 40 cm horizontal bore magnet. The system is equipped with a 200 mT/m gradient system. Examinations were conducted using a 72-mm birdcage resonator for excitation, and detection was achieved using a 3 cm surface coil. An initial sagittal scout image was obtained in order to reproducibly localize transverse sections from the cerebellum to the olfactory bulbs. Ten transverse and thirteen sagittal sections of T2-weighted spin echo images were acquired consecutively using a rapid-acquisition relaxation-enhanced sequence (RARE) with the following parameters: TR, 4075 ms; slice thickness, 1 mm; distance between slices, 0.2 mm; field of view, 35×25 mm; matrix, 256×192; and number of averages, 8. For the transverse plane, a TE value of 100 ms was used to facilitate detection of abnormal T2 signal, while a TE value of 40 ms was used to improved conspicuity of the corpus callosum in the sagittal plane. The total scan time was about 12 minutes for each transverse and sagittal set of MR images. Volumetric analyses of the corpus callosum were performed on sagittal MR images using Bruker image processing and analysis software. Total volumes were computed by combining the results from a series of ten MR imaging slices, with the resulting data expressed as mean values.

Animals were housed and cared for in accordance with the National Institutes of Health (NIH) guidelines for animal welfare and all animal experiments were performed in accordance with protocols approved by our Institutional Animal Care and Use Committee (IACUC).

## Results

### Whole brain irradiation permanently decreases the number of proliferating cells in the SVZ, the corpus callosum and the cortex

Animals received a single dose of whole brain X-irradiation (25Gy) at age 3 months and were sacrificed at various time points following administration ranging from 24 hours to 15 months. Data were compared to untreated age-matched control animals. All animals (irradiated and control group) were injected with BrdU for 3 consecutive days just prior to sacrifice.

**Figure 1:**
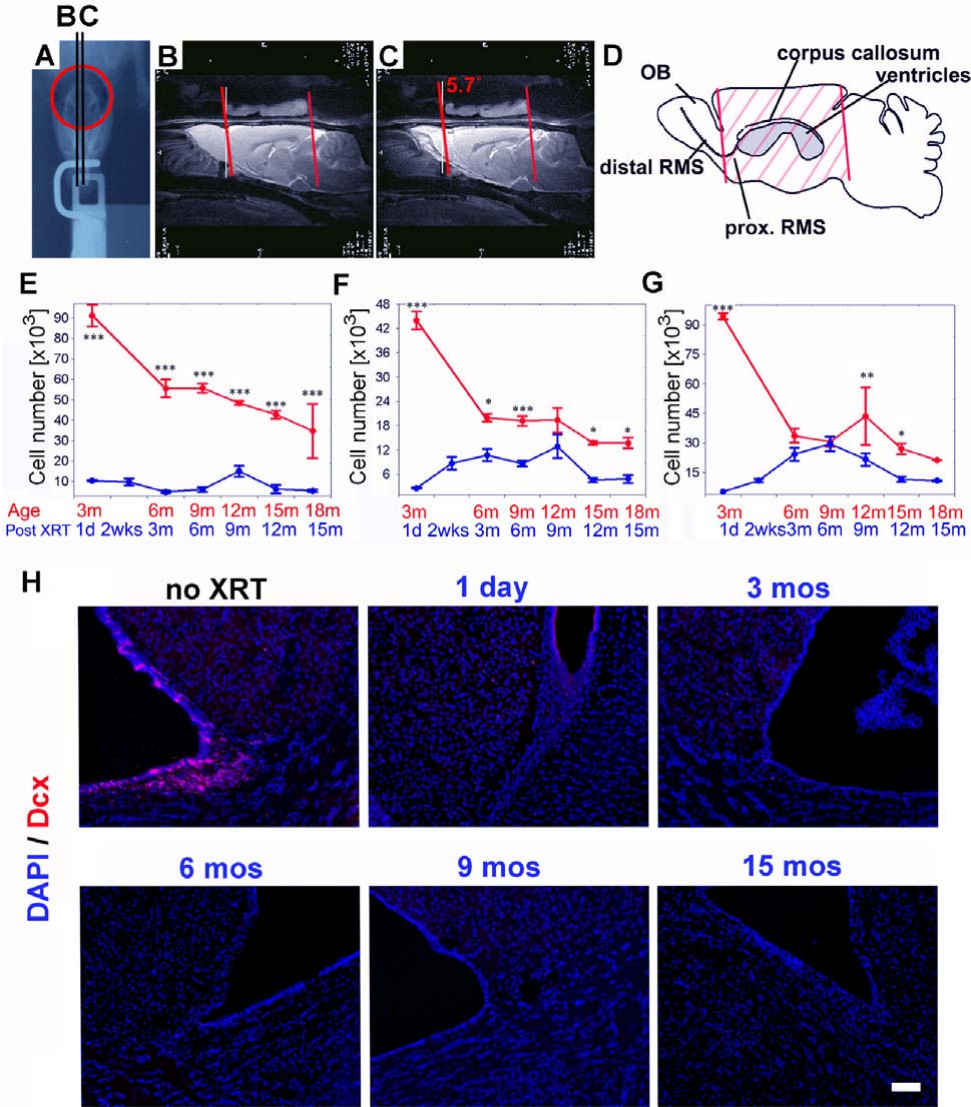
Experimental set-up and definition of radiation field as demonstrated in a representative double-exposure X-ray of the skull of a rat secured in the radiation device (A). The red circle indicates the opening in the radiation shield and the skull components that are exposed to radiation. MRI detectable gel polymer (IZI Medical Products, Baltimore MD) was placed over the area defined by the shield opening and sagittal MRI images performed (B, C) in order to demonstrate the brain volumes exposed to radiation. Red lines demonstrate the path of the radiation beam, at 5.7 degrees from the vertical. This is represented schematically in (D) to clearly illustrate the exclusion of the olfactory bulb and the distal most portion of the RMS from the radiation field. Stereological estimates of absolute BrdU counts in the SVZ, corpus callosum (CC) and cortex (Cx) (E–G) in irradiated (blue) and normal aging (red) rats. There is significant suppression of proliferation on day 1 post radiation in all 3 regions. BrdU levels are most significantly suppressed in the SVZ. In the cortex there is recovery to age-matched control levels at 3–6 months post radiation. In parallel, doublecortin labeled neuroblasts in the SVZ (H) are completely suppressed as of day 1 post radiation and do not recover up to 15 months post XRT. Stars indicate statistical significance (*** p<0.001; ** p<0.01; * p<0.05; ANOVA). Bars = SEM. Scale bar = 100 µm.

The radiation set-up was designed to deliver whole brain radiation excluding the olfactory bulbs which were covered by lead shielding (see methods and Figure 1A–1D). Sparing of the olfactory bulbs was confirmed by a double exposure X-ray of the skull prior to each radiation exposure (Fig. 1A). We also performed magnetic resonance imaging (MRI) on representative animals after covering the opening in the lead shield with MRI-sensitive material (Figure 1B–1C). Both imaging modalities served to confirm that the entire telencephalon was included in the radiation field to the exclusion of the olfactory bulbs. Scatter at the edge of the lead shield is considered minimal.

Using stereological methods (optical fractionator, Stereo Investigator, Microbrightfield, Vermont) we quantified the total number of BrdU+cells in three brain regions (SVZ, cortex and corpus callosum) at about 3 month intervals. The total number of BrdU+cells in the SVZ was significantly decreased one day after radiation (91,039+/−3,783 prior to irradiation, 10,469+/−311 one day after radiation). The number of BrdU+cells in the SVZ remained suppressed throughout the entire period of analysis without an obvious attempt at recovery (Figure 1E). At 15 months post radiation, corresponding to 18 months of age, the average number of BrdU+cells in the SVZ was 5,541+/−624 compared to 34,680+/−9,413BrdU+cells in age-matched control animals. Statistical analysis confirmed a significant decrease in BrdU+cells in the SVZ of irradiated animals compared with control animals at all points examined (ANOVA, p<0.05, Newman-Keuls). In addition to the suppression of proliferating cells, the SVZ looses all its doublecortin (DCX)-expressing neuroblasts permanently as of day 1 and up to 15 months post radiation (Figure 1H).

In the cortex and the corpus callosum (Cx and CC respectively), the initial decline in the number of proliferating cells (one day post XRT) was even larger in magnitude compared with that in the SVZ (16-fold decrease in BrdU+cells in the CC, 17-fold in the Cx and 9-fold in the SVZ). However, unlike in the SVZ, both Cx and CC demonstrate a transient increase in BrdU+cells over time. This was most evident in the cortex whereby at 3 months post XRT, the irradiated animals exhibit essentially the same number of proliferating cells compared with normal age-matched controls. This “recovery period” is maintained up to 6–9 months post XRT, but is not sustained at later time points (Figure 1F–1G). This data suggests that the local pool of proliferating cells is capable of compensation for acute cell loss for a fairly sustained period; its failure at a late time point may be related to the absence of input from the stem cell compartment in the SVZ.

### The olfactory bulb exhibits sustained but non-permanent recovery of neurogenesis despite complete SVZ suppression by radiation

**Figure 2:**
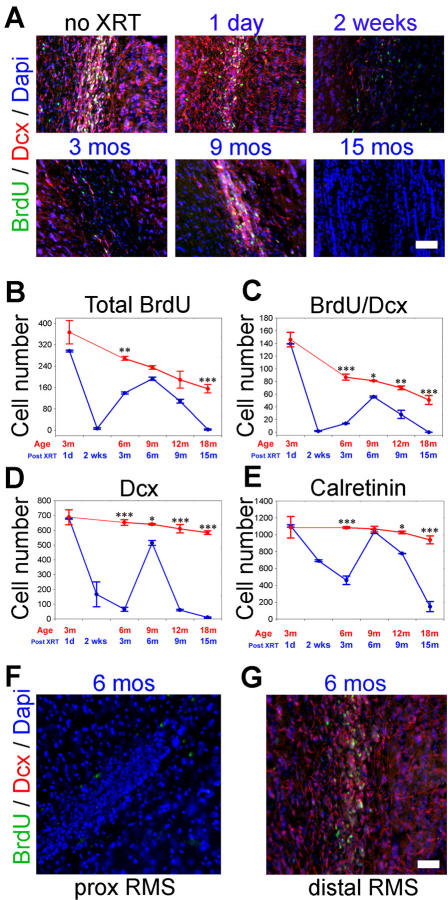
Effect of radiation on the olfactory bulb. (A) Immunohistochemistry of BrdU/doublecortin (DCX) labeled cells shown in cross sections of the olfactory bulb at progressive time points post radiation. Quantitative measurements of total BrdU cells, BrdU/doublecortin in the distal RMS, doublecortin cells in the granular cell layer (DCX) and periglomerular calretinin-expressing interneurons are shown in B–E. Suppression of BrdU and DCX cells is delayed to 2 weeks post radiation but is followed by an immediate attempt at recovery peaking at 6 months post XRT. The origin of this recovery is thought to be due to proliferating neuroblasts in the distal RMS just proximal to the olfactory bulb. Concomitantly the SVZ and proximal RMS are devoid of proliferative activity and DCX expressing neuroblasts (F–G). Stars indicate statistical significance (*** p<0.001; ** p<0.01; * p<0.05; ANOVA). Bars = SEM. Scale bar in (A) corresponds to 50 µm; in (F) and (G) to 35 µm.

We next addressed the impact of radiation-induced loss of BrdU+cells in the SVZ on olfactory neurogenesis. To this end we quantitated the number of BrdU+cells in the entire olfactory bulb (OB), the number of BrdU+/DCX+cells in the rostral migratory stream (RMS), the number of neuroblasts (immunostained for doublecortin, DCX) in the granular layer of the OB, and the number of mature interneurons (calretinin+cells) in the glomerular layer. Twenty-four hours after irradiation, BrdU+cells in the olfactory bulb (OB), excluded from the radiation field in our model, are unaffected (Figure 2A–2B). However, by two weeks after irradiation BrdU+cells in the OB and proliferating neuroblasts (BrdU/DCX+) decrease dramatically in numbers to 2% and 1.3% of control levels respectively (Figure 2B–2C). The delayed loss in proliferating cells likely reflects the absence of incoming neuroblasts due to the suppression of SVZ neurogenesis. The total number of doublecortin+cells in the granular cell layer declines to 24% of control levels at two weeks (Figure 2D). In the normal rat OB, these cells migrate radially to give rise to glomerular olfactory interneurons. In contrast, the number of fully differentiated Calretinin+olfactory interneurons in the glomerular cell layer exhibits only a moderate decline at two weeks post-radiation to 62% of control levels (Figure 2E). The relative sparing of the Calretinin+interneurons at two weeks probably reflects the slower turn over rate of mature interneurons in the OB compared with the DCX+cell compartments.

Interestingly, despite the persistent suppression of the SVZ, robust proliferation resumes in the olfactory bulb over time. At 3 months post radiation, the total number of BrdU+cells in the OB is up to 52% of control levels and continues to increase up to 6 months after irradiation, reaching near control levels (Figure 2B). Similarly, the number of proliferating DCX+neuroblasts increases to 16% of control levels at 3 months and reaches near control levels by 6 months after irradiation (Figure 2C). Calretinin+neurons continue to decline in number for the first 3 months after irradiation but rebound back to near control levels by 6 months. The robust recovery of proliferating cells, neuroblasts and interneurons in the OB at 6 months after irradiation is remarkable considering a complete lack of recovery in the SVZ for both BrdU+and doublecortin+cells at the same time point (Figure 1C and Figure 1H). Systematic analysis of BrdU+cells along the RMS demonstrates this surge of proliferating cells to persist from the OB back into the distal RMS. A sharp transition into a region of complete absence of BrdU uptake follows, corresponding to the margin of the radiation field at the proximal RMS (Figure 2F–2G). Nonetheless, the recovery at 6 months after irradiation is not sustained long-term. All cell subpopulations subsequently exhibit a dramatic decline in numbers over time. By 15 months after irradiation, proliferating DCX+cells are absent in the olfactory bulb of irradiated animals and the total number of BrdU+cells in the OB declines sharply to 1.6% of control levels. Granular layer DCX+neuroblasts drop to 2% and calretinin+olfactory interneurons decline to 15.9% of control levels.

### Radiation results in a progressive decline of oligodendrocyte precursor cells and late widespread demyelination

We evaluated the impact of radiation on the number of proliferating and mature oligodendrocytes at various time points after irradiation. We quantified the number of BrdU+/NG2+cells in multiple brain regions and the expression of O4 in the corpus callosum as indicators of oligodendrocyte progenitors. We also quantified the expression of MAG (myelin associated glycoprotein) and MBP (myelin basic protein) as markers of mature oligodendrocytes and myelination parameters. The diffuse pattern of immunostaining for O4, MAG and MBP prohibits accurate stereological cell counts. We thus performed fluorescence image intensity analysis for each of these markers using NIH Image software (see material and methods for technical details).

We demonstrate significant and permanent suppression of proliferating NG2 cells in the SVZ immediately after radiation down to 8% of control levels, with a small but statistically insignificant rise at 9 months post treatment (Figure S1). The response in the corpus callosum and cortex differs significantly, as the proliferating NG2 cells exhibit an initial steep decline followed by a fast and successful compensatory response that results in near normal cell numbers by 6 months post XRT in the cortex with a similar but less extensive response in the corpus callosum (Figure S1C–D). Interestingly, NG2+cycling cells decrease steadily with aging in the normal animal. In the irradiated brains, the number of proliferating NG2 cells decreases again at 1 year and at 15 months post XRT but is not significantly different from normal age-dependent decline.

**Figure 3:**
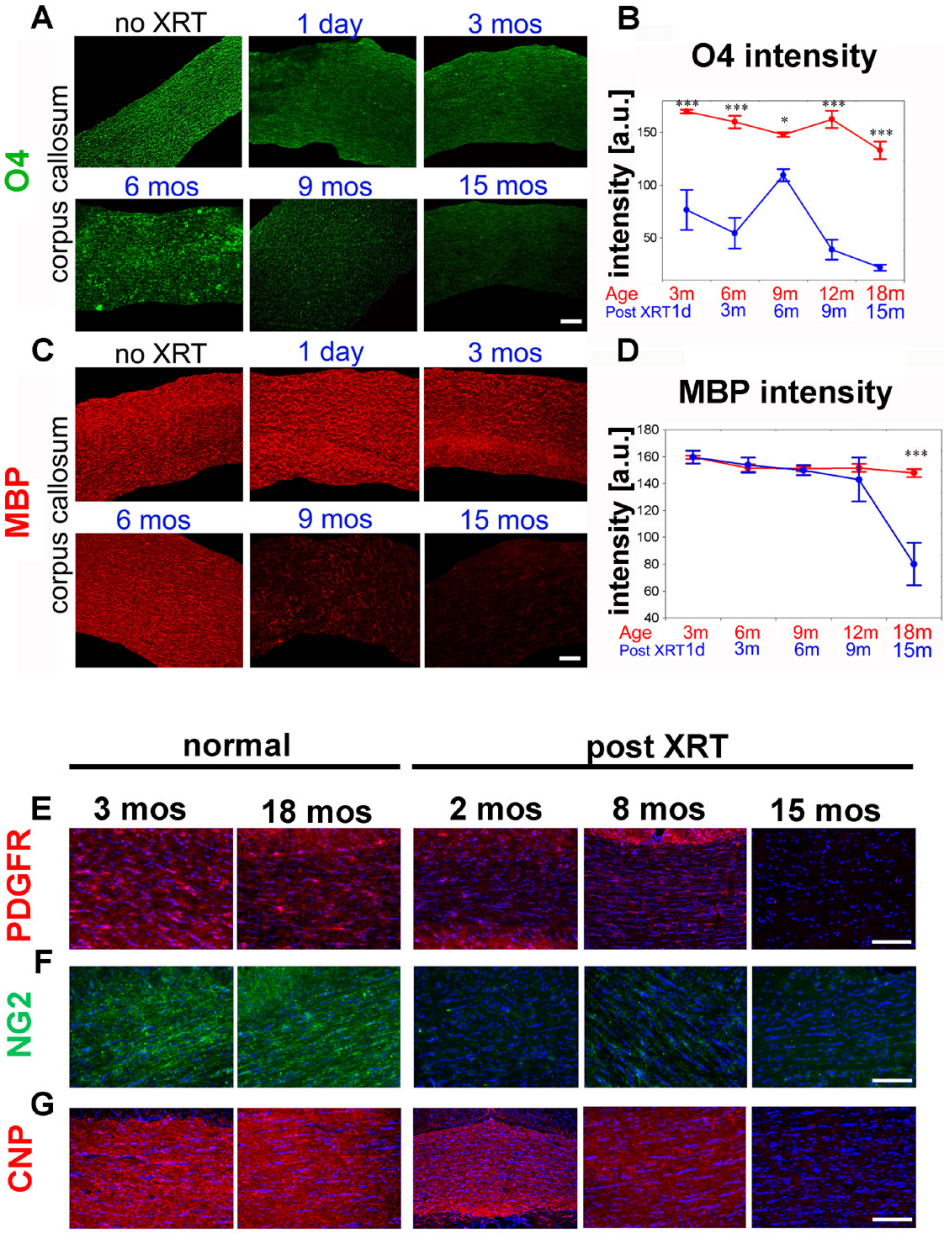
Fluorescence intensity quantification of O4 and MBP and immunohistological assessment of oligodendrocyte markers. (A)Representative contoured sections from the genu of the corpus callosum at serial time points post radiation immunostained for O4 (green). Quantitative measurements are shown in (B). A steady decline in O4 expression is seen immediately following administration of radiation until 3 months post XRT. At 6 months post radiation, a spike in O4 levels is followed by a significant decrease that persists until one year and thereafter. By 15 months, the majority of O4+cells are depleted. In comparison, aged control animals maintain a steady level of O4 until 18 months of age. (C) Serial immunohistochemical stains for MBP (red) on representative sections from the genu of the corpus callosum at various time points post radiation. Quantitative measurements are shown in (D). MBP expression is sustained until 6 months post radiation. By 9 months, patchy loss of myelin is observed throughout the corpus callosum. Demyelination is widespread by 15 months. Oligodendrocyte precursor markers, PDGFR (E) and NG2 (F) exhibit no significant change in intensity in aging animals but decrease rapidly after radiation without recovery, up to 15 months later. Markers of more mature oligodendrocytes such as CNP (G) exhibit a delayed decrease in expression starting at 8 months post XRT. CNP is significantly depleted at 15 months post XRT. DAPI in blue. (*** p<0.001; ** p<0.01; * p<0.05; ANOVA). Bars = SEM. Scale bar corresponds to 100 µm in all panels.

The impact of irradiation on oligodendrocyte precursor cells was further examined by quantitative fluorescence imaging for O4. We observed a steady decline in O4 signal starting one day after radiation and reaching about 30% of control levels by 3 months. At 6 months there is a surge in O4 levels followed by a more significant decline at one year and thereafter. The aging normal animals maintain a very steady level of O4 at least until 18 months of age despite decreasing levels of proliferating NG2 cells. This suggests highly controlled and efficient regulation of the O4 cell subpopulation (Figure 3A–3B).

MBP, a marker of mature oligodendrocytes, exhibits a different pattern. It remains essentially unchanged for the first 9 months following irradiation. However, later time points show a significant decrease in MBP image intensity to levels corresponding to 54% of control values by 15 months (Figure 3C–3D). Histological examination of tissue sections demonstrates a diffuse pattern of myelin loss throughout the corpus callosum as well as the fimbriae, the external capsule and the deep white matter. Similar results were observed when quantifying image intensity for MAG (data not shown).

We also analyzed other markers of the oligodendrocyte lineage that cover the early, intermediate and more mature stages of oligodendrocytic differentiation. PDGFRA and NG2 followed a pattern very similar to O4 with early loss and no recovery (Figure 3E–3F); CNPase decreased measurably in later time points post XRT (Figure 3G) as did O1 and MAG (Figure S2) with perhaps an earlier onset of O1 loss. The expression pattern of these markers is compatible with early loss of immature oligodendrocyte precursors and delayed loss of more mature progeny, confirming our stereological and intensity quantification analyses.

Areas of patchy necrosis and focal total demyelination with significant cell loss are seen only beyond 15 months post XRT in about 30% of all animals allowed to live up to that time point (n = 9) (Figure S4). The incidence of necrosis post XRT in this study is similar to what is reported in the literature [19].

### Magnetic resonance imaging demonstrates early reduction in corpus callosum volume and T2 changes correlating with loss of progenitors and demyelination

**Figure 4:**
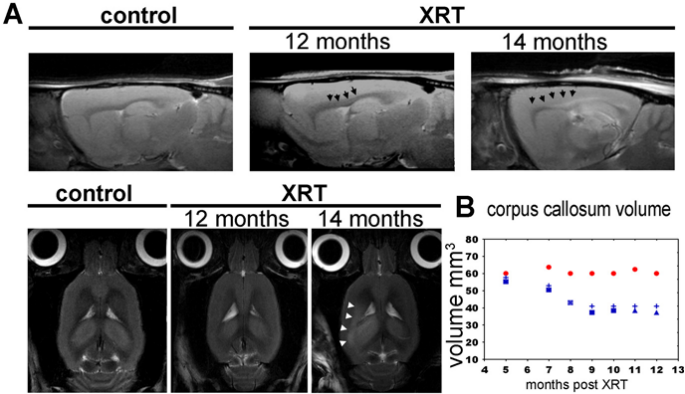
MRI imaging post radiation. Representative sagittal (A, upper panel) and axial (A, lower panel) images of control or irradiated rats. At 12 months, subtle T2 changes are seen in the corpus callosum (arrows) that correspond to demyelination changes observed histologically. At 14 months post radiation, the T2 signal changes are more definite. (B) Graph depicts changes in the corpus callosum volume in irradiated animals as compared to aging control.

**Figure 5:**
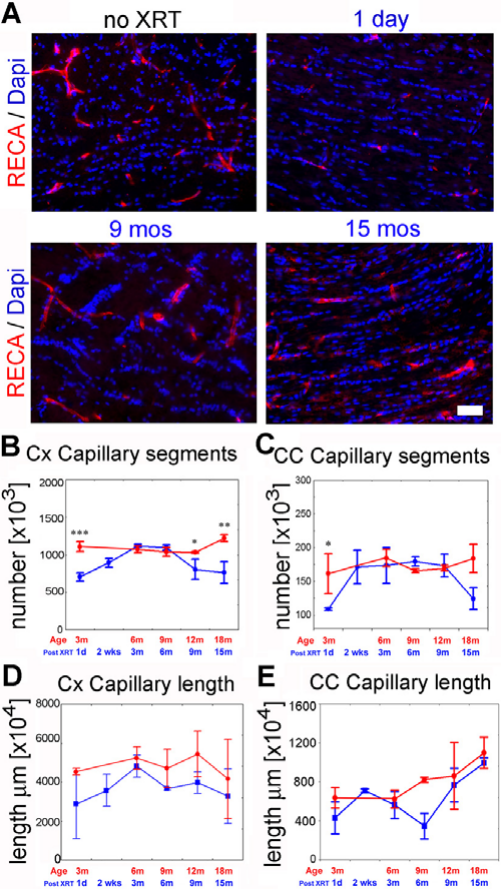
Endothelial cell number and capillary length post radiation. (A) Representative images of sections from the corpus callosum immunostained for rat endothelial cell antigen (RECA) at various time points post radiation. RECA expression declines immediately post radiation but is restored and maintained through 15 months. Stereological estimates of the number of capillary segments in the cortex (B) and corpus callosum (C) and of capillary length in both regions (D, E). (*** p<0.001; ** p<0.01; * p<0.05; ANOVA). Bars = SEM. Scale bar in A corresponds to 100 µm.

Serial T2-weighted MR imaging was performed on irradiated and control animals in order to detect structural or signal changes that may correlate with the histological findings above. Description of MRI findings in the literature often pertains to supra therapeutic doses and very late changes associated with necrosis. Here we follow animals with monthly scans starting at 5 months post radiation and spanning a period of 9 months (Figure 4). No signal changes are noted in the early and intermediate phases post radiation. By 12 months, subtle T2 signal increases are seen within the periventricular white matter and corpus callosum suggestive of demyelination as seen concomitantly by immunohistological examination. Progressive thinning and loss of definition of the corpus callosal margins, primarily along the body, are difficult to detect qualitatively until 13 months. However, serial volumetric measurements of the callosal contours identify definite loss of volume starting as early as 5 months post XRT (Figure 4B). These changes in volume are seen well before significant demyelination is identified by histology or MRI, and could possibly be related to the significant loss of oligodendrocyte precursors. An increase in the size of the ventricular system is also identified as a function of time following radiation treatment, and could be attributed to similar cell losses in the brain parenchyma. Later time points demonstrate worsening T2 signal abnormality within the periventricular and deep cerebral white matter structures, the external capsule, and the fimbriae (white arrows, Figure 5). These imaging features precede histological findings of patchy demyelination and necrosis seen beyond 15 months (Figure S4).

### Endothelial cell number declines immediately following high dose X-irradiation but is restored to control levels as early as two months after exposure

Endothelial cells are recognized targets of early radiation-induced apoptosis and are likely involved in delayed vascular necrosis. Some authors have implicated endothelial cell damage as a prime element in the pathogenesis of demyelination. Here we serially follow the number of capillary segments for 15 months post radiation. Using immunostaining for rat endothelial cell antigen (RECA, Figure 5A) and previously described stereological methods[17], [18], [20], we calculated the total number of capillary segments as well as capillary length in the cortex and the corpus callosum. The total number of capillary segments decreases significantly one day after radiation in both the CC and Cx by 33% and 36%, respectively (Figure 5B–5C). This is followed by rapid recovery to normal levels, particularly in the corpus callosum which recovers in 2 weeks. The number of vessels remains steady until about 15 months post radiation when it decreases again, reaching a statistically significant difference in the cortex only. Capillary length was estimated using the virtual sphere probe method (Stereo-Investigator, Microbrightfield, VT)[17] and was found to be lower than normal throughout the entire period studied, but never reaching a statistically significant difference from age-matched control (Figure 5D–5E).

### Histological assessment of patient-derived irradiated white matter reveals early loss of oligoprogenitor markers and delayed disappearance of myelin

**Figure 6:**
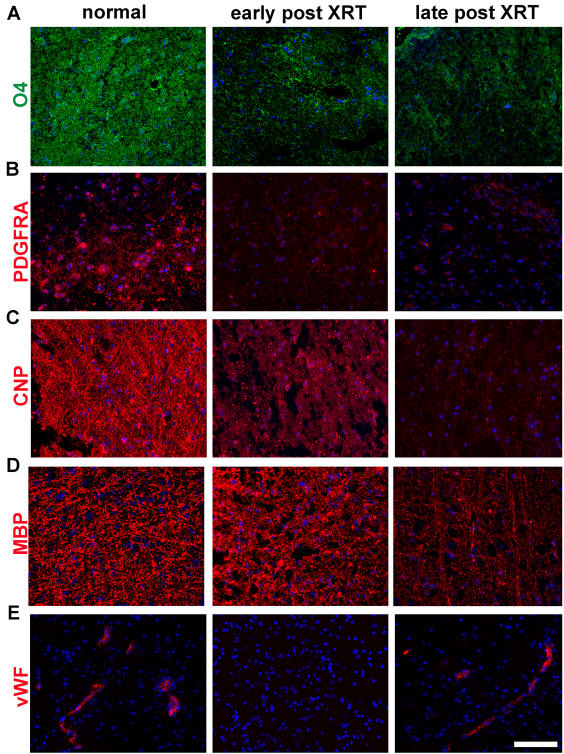
Radiation effects in human tissue samples. Human white matter samples acquired from non-irradiated brain (normal), irradiated specimens up to 7 months post XRT (labeled “early”), and irradiated specimens beyond 9 months up to 7 years (labeled “late”). Immunohistochemistry for early oligodendrocyte progenitor markers (O4, PDGFR), more mature oligoprogenitors (CNP) and fully differentiated oligodendrocytes (MBP) and endothelial cells (von Willebrand factor, vWF). Human tissues exhibit a pattern of early loss of young oligodendrocyte progenitors and delayed loss of more mature oligodendrocyte lineage cells, similar to what was described in the irradiated rat brain. Endothelial cells are scarce in early post radiation tissues and commonly normal in number in late post XRT tissues (7 years in this panel). DAPI in blue. Scale bar corresponds to 100 µm in all panels.

Irradiated human tissues are difficult to obtain due to the relative infrequence of surgical intervention after irradiation and the absence of adequate annotation of tissues obtained from large tissue banks. In addition, such samples have to be meticulously acquired in order to avoid contamination by neighboring tumor tissue. Our tissues were collected in accordance with federal and institutional guidelines and following IRB approval. The majority of samples consisted of subcortical white matter resected in the periphery of a brain metastasis or a meningioma. These tumors are usually non-infiltrating and surrounding brain tissue is removed occasionally as part of standard neurosurgical techniques to allow access to the lesion. Patients with CNS metastases present to surgery soon after diagnosis or upon recurrence following the administration of radiation. Most patients received radiosurgery which consists of high dose focal irradiation (18–21 Gy). Normal control consisted of white matter tissue surrounding a lesion in the context of a previously untreated patient. Both “normal” and “irradiated” tissues are likely to have exhibited a degree of edema, as is common in brain tissue surrounding a neoplastic process. A total of 7 normal controls and 11 irradiated samples ranging from 2 months to 7 years post radiation were collected over 3 years. Two of the irradiated samples were obtained due to clinically relevant “radiation necrosis” and were confirmed upon pathological analysis to represent frank necrosis and no tumor. Those samples were analyzed separately (Figure S4). We grouped samples dating up to 7 months post XRT under “early/subacute” and those obtained at longer intervals (9 months to 7 years post XRT) as “Late”. Mean patient age was 55 years in the non-irradiated control group and 56.2 and 52 years in the “early” and “late” groups respectively. Compared to “normal brain”, irradiated samples exhibit evidence of early loss of O4 and PDGFRA expressing oligoprogenitors (as early as 2 months post XRT, our earliest time point) (Figure 6A–6B), that persisted up to several years post treatment. Markers of intermediate stages of differentiation (O1 and CNP, Figure 6C and Figure S2) were reduced at later time points but also remained suppressed throughout the observation period (up to 7 years). More mature markers such as MBP (Figure 6D) and MAG (Figure S2), in addition to Galactocerebroside (Galc), remain strongly expressed at early time points post-radiation but decline dramatically over time beyond a year after exposure (Figure S2). We also evaluated capillaries by immunostaining for von Willebrand Factor (vWF, Figure 6E) and found a trend similar to what is seen in the rat with early loss of endothelial cells but a more significant presence of endothelial cells and capillaries at later time points. A quantitative study could not be undertaken in view of the small number of tissues and the wide range of doses and times post XRT but the trend of early loss of oligodendrocyte progenitors and endothelial cells was definite in all tissues examined. Also highly consistent was the near absence of oligodendrocyte progenitors in all late tissues examined (up to 7 years post XRT). Endothelial cells clearly were present in late tissues, although we could not assess capillary complexity or branching. Loss of myelin and preservation of axons was also very consistent in late tissues in both rats and humans (Figure S3).

**Figure 7:**
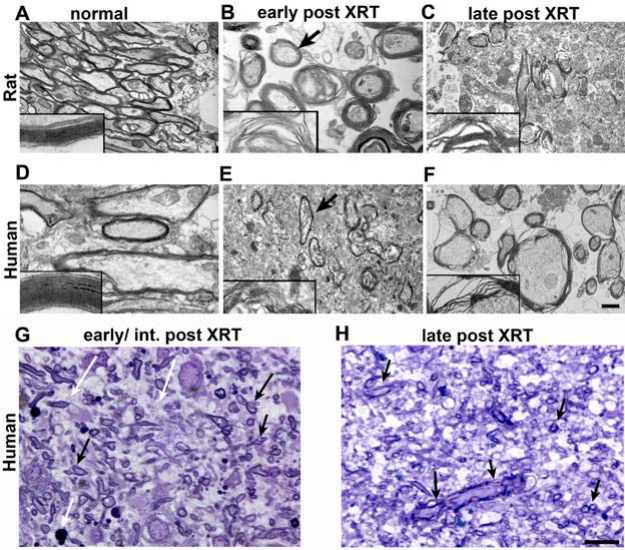
Ultrastructural features of irradiated rat and human brain tissue. Electron microscopy of rat (upper panel) and human (lower panel) tissues in normal controls (A, D), an early/intermediate time point post radiation (B, E, 11 months and 7 months, respectively) and a late time point (15 months in the rat (C) and 7 years in the human (F)). Ultrastructural analysis of the myelin sheaths demonstrates normally compacted lamellae in the normal brains. At about 7 months post XRT, myelin sheaths in both human and rats (B and E and insets) acquire an irregular appearance with segmental loss of lamellar compaction associated with separation at the intraperiod line. These changes are more prevalent in larger myelin sheaths and are often mixed with normal myelinated fibers (arrows in (B) and (E)). Later times post XRT are associated with an increasing frequency and severity of myelin sheath degradation and vesiculation. Insets are magnifications of representative areas of myelin sheaths in each panel. Semithin toluidine sections of irradiated human tissue are shown in (G) and (H). Evidence of myelin sheath fragmentation is seen in the early/intermediate time point (14 months post XRT in (G)) as well as cytoplasmic lipid debris (white arrows) suggestive of active myelin degradation. Abnormal myelin sheaths persist and occur at higher frequency at late time points (7 years in H). Scale bar in (F) corresponds to 0.85 µm in (A), (D) and (E), 0.3 µm in (B) and (F) and 0.5 µm in (C). Scale bar in (H) corresponds to 10 µm in (G) and (H).

Electron micrographs of irradiated rat and human tissues showed a very similar process of degradation of the myelin sheaths, which acquire an irregular appearance with segmental loss of lamellar compaction associated with separation at the intraperiod line (Figure 7A–7F). The axons within the myelin sheaths appeared normal with appropriate orientation of microtubules and intermediate filaments and only occasional dense bodies; no spheroids or filamentous aggregates were seen. Myelin changes were patchy in nature and mixed with normal appearing myelin sheaths in the same regions. As time post XRT progressed, an increasing number of fragmented myelin sheaths was observed, primarily surrounding larger axons. Ultrastructural changes suggestive of axonal damage were seen within myelin sheaths exhibiting significant fragmentation and vesiculation at late times post XRT, but overall evidence for injury to neuronal cell bodies was scarce. Scattered fibers possessing inappropriately thin myelin sheaths for their axonal diameter were noted. In the two cases where white matter was resected specifically due to symptomatic radiation necrosis, areas of acellular amorphous debris and hyalinization of blood vessels could be seen (Figure S4). Examination of tissues stained in toluidine blue demonstrated a progressive degeneration of the myelin sheaths over time, associated with a moderate number of microglia or macrophages containing cytoplasmic lipid (Figure 7G–7H). An assessment of neuronal and oligodendroglial counts using toluidine semithin sections could not be performed due to lack of normative data necessary to control for regional differences in cell distribution between tissues obtained from different sites.

## Discussion

The main findings of this study pertain to the dramatic and irreversible suppression of subventricular zone neurogenesis and the loss of oligodendrocyte precursors following whole brain radiation. There was no significant recovery in the SVZ up to 15 months post radiation. The irreversible and progressive loss of proliferating cells in the SVZ could be due to a loss in stem cell numbers or the functional inactivation of the stem cell pool. Our data stand in marked contrast to data based on pharmacological suppression using antimitotic agents such as Ara-C [21]. SVZ exposure to AraC leads to a dramatic but transient loss of all precursors (type A and C cells). The relatively quiescent SVZ astrocytes (type B cells) remain largely intact, reenter the cell cycle after washout of AraC and repopulate the entire SVZ within a week [21].

Previous studies have suggested that radiation can suppress proliferating SVZ cells for at least 3 months [12]. Our data extend these observations for up to 15 months confirming that SVZ damage is truly irreversible. Interestingly, regions outside the SVZ such as the corpus callosum and the cortex are capable of at least partial recovery. An increase in proliferating cell numbers is seen in both regions within 2 weeks post radiation. This recovery approaches age-matched control cell numbers, particularly in the cortex, but is not sustained beyond 9 months post XRT. Such a transient proliferation response is compatible with the activation of neural precursors with limited self-renewal potential resulting in a transient recovery followed by exhaustion of the precursor pool. It also implicates the loss of long-term self-renewing stem cells or their inability to re-enter the cell cycle.

One particular feature in our experimental design was the shielding of the olfactory bulb from radiation exposure. The response in the OB is essentially tri-phasic. There is initial loss of neuroblasts coming in from the SVZ with delayed loss of glomerular calretinin-expressing neurons. A second phase involves a robust recovery characterized by neuroblast proliferation and leading to a successful and complete repopulation of the glomerular neurons by 6 months post XRT. This occurs despite continued suppression of proliferating cells and complete absence of DCX+cells in the SVZ and proximal RMS. This proliferative activity is initiated in the distal RMS, which was effectively shielded from high dose irradiation and is likely due to the ability of local neural precursors to self-renew and repair their niche independently of the SVZ[22]. Nonetheless, the recovery fails dramatically beyond one year, with complete exhaustion of proliferating doublecortin cells and significantly reduced calretinin neurons. We hypothesize that this result is due to the continued suppression of the SVZ and the lack of long-term renewing precursors in the RMS and OB. Therefore high dose radiation resulted in greater suppression of the quiescent SVZ stem cell compartment compared with the cycling progenitor populations outside the SVZ. Additional regional influences may also play a role since the same cell populations (BrdU/NG2) follow different kinetics depending on their location, with greater and permanent suppression experienced in the SVZ, compared to the cortex or callosum. An alternative explanation for this finding is a region or niche-dependent difference in stem cell or precursor origin.

Previous studies suggested that niche-dependent inhibition of stem cell function is responsible for the reduction in hippocampal neurogenesis observed after radiation [23]. While the OB here was shielded from the direct effects of radiation, it could have been affected by a bystander effect [24]. This phenomenon is considered an important mediator of the delayed effects of radiation and is typically transmitted via cytokine secretion or intercellular contacts such as gap junctions. In our study we cannot rule out that perturbations in the OB niche occurred in a delayed fashion. However this appears to be an unlikely interpretation of the data since the decrease in calretinin neurons is accompanied with a decrease in both total and proliferating doublecortin+neuroblasts. Our data strongly suggest that neuron loss in the OB is dependent on SVZ precursor cell loss rather than niche related changes in the OB.

Outside of the SVZ and hippocampal progenitor pools, the long-term effects of brain irradiation are poorly understood. In the normal brain, the majority of cycling cells are thought to be NG2-expressing oligodendrocyte progenitors. While there is data suggesting they may have multiple functional roles within the adult CNS[25], NG2+cells are considered part of the oligodendrocyte lineage and are capable of giving rise to new oligodendrocytes under both normal and demyelinating conditions[26]–[28]. During differentiation, NG2 cells, often co-expressing PDGFRA, are gradually downregulated and cells enter a transitory pro-oligodendrocyte stage where they express the O4 antigen. As cells mature, they progressively lose expression of progenitor markers and acquire markers of mature oligodendrocytes, including MBP, MAG and CNP[29], [30]. Other data show that NG2 cells may be recruited to a demyelinated area[31], [32] but do not always contribute to efficient remyelination. These studies suggest that environmental factors play a significant modulatory role that may inhibit NG2 cell differentiation. The interpretation of our NG2 findings is complicated by the effect of aging whereby proliferating NG2 cells decrease steadily especially beyond a year of age. There are also regional differences, with the cortex and callosum exhibiting some recovery of NG2 proliferation following radiation, but not the SVZ. This could indicate context dependent alterations in NG2 cell behavior or fate. More recently lineage tracing studies have shown that the adult SVZ can contribute to oligodendrogenesis [33], [34]. Despite questions about the fate of NG2 cells and their pleomorphic role and in light of the concomitant loss of PDGFRA and O4 it is reasonable to conclude that the depletion of cycling NG2 cells contributes to the inability to remyelinate.

Our data suggest that normal animals have the ability to maintain O4 levels in aging despite a decrease in cycling NG2 precursor cell numbers. In contrast irradiated animals are incapable of maintaining O4 levels either due to loss of NG2 precursors below a critical threshold or loss of the mechanism that controls O4 homeostasis. The robust recovery response of the NG2/BrdU+precursors to near normal levels argues against NG2 precursor cell loss as the primary reason for the inability to maintain O4 levels post radiation. However at late time points (beyond 1 year post XRT) the number of NG2 BrdU+cells may be below a potential critical threshold required for replenishment of the O4 pool. In normal animals, a relatively small number of cycling NG2 cells (30% of 3-month control animals) is sufficient to maintain O4 levels during aging while irradiated animals with similar NG2/BrdU cell numbers are unable to sustain O4 levels. The factors that control O4 levels for a given number of oligodendrocyte precursors are not known but may include cell autonomous or environmental factors that impact progression along the oligodendrocyte lineage. Alternatively NG2 progeny may not survive due to radiation-related mitotic cell death or to the perpetuation of cytokine cascades triggered by tissue response to XRT[35].

The response of MBP expressing cells is unique among all the cell populations described here. In contrast to the oligodendrocyte precursor markers such as NG2/BrdU, PDGFRA or O4, MBP in irradiated animals was maintained at close to control levels until 9 months post radiation. However, beyond 1 year we observed a rapid decrease in MBP. Late onset demyelination after brain irradiation has been described in multiple species including humans[36] but the mechanism for this delayed response has remained unclear[37]. One possible explanation is a tight control of MBP levels despite a significant decrease in oligodendrocyte precursor cells. The lack of an initial MBP loss suggests that MBP producing cells are relatively resistant to the immediate effects of radiation presumably due to their highly differentiated nature. While the exact turn-over rate of mature MBP+cells is not known, the kinetics of MBP loss after radiation is compatible with a very slow turn over rate keeping MBP at near normal levels for up to 12 months. The loss of MBP levels beyond the putative MBP turn-over rate could not be compensated due to the lack of functional oligodendrocyte precursors. Alternatively, MBP turn-over rates may be faster and MBP levels actively maintained through proliferation and differentiation of the oligodendrocyte precursor cell compartment. In such a scenario loss of oligodendrocyte precursors below a critical threshold or an inability to maintain MBP homeostasis may trigger late onset MBP loss. Ultrastructural studies demonstrate clearly that MBP levels are not only downregulated but are associated with structural damage to the myelin sheath indicative of oligodendrocyte death or dysfunction. The failure of recovery could be due to the transmission of radiation-induced genetic instability over many cell divisions leading to delayed reproductive death of cells in the oligodendrocyte lineage [38].

Some authors have attributed demyelination to endothelial cell damage, ischemia and necrosis[39]. In fact, endothelial cells have been invoked as the primary target of radiation to the CNS as they are sensitive to acute radiation damage. However there is limited information about the long-term effects of radiation on endothelial cell numbers[40] . Here we report that endothelial cell numbers recover to near control levels within 2 weeks and remain within normal range for periods beyond onset of demyelination. In recent work Hopewell's group[41] demonstrated that radioprotection of endothelial cells against apoptosis reduces the risk of delayed radiation-induced necrosis but did not comment on the impact of radioprotection on demyelination. There are additional recent investigations that suggest that depletion of precursors is independent of damage to the vasculature.[42]. Here we demonstrate demyelination by radiographic and histological methods prior to the occurrence of vascular necrosis at a stage when endothelial cell numbers are close to normal levels. Furthermore, demyelination occurs in a diffuse histological pattern while necrotic events, observed several months after onset of demyelination, occur in focal areas, particularly in the corpus callosum and fornix. It is important to note that the study of the vascular system here is purely structural. Changes in endothelial cell function such as capillary permeability and status of VEGF pathways have not been investigated and may still play a role in facilitating demyelination[39].

Interestingly our data in the rat model were further corroborated by the analysis of clinical specimens of human brain at early and late time points post radiation. An early loss of oligodendrocyte precursors, as evidenced by loss of O4 and PDGFRA expression, preceding demyelination and a near complete recovery of endothelial cell numbers supports the hypothesis that loss of oligodendrocyte precursors is a primary event. Additionally, electron micrographs of human and rat specimens at various time points after radiation support our findings by revealing a similar pattern of myelin sheath degradation over time post radiation with absence of significant axonal damage. This pattern of loss of lamellar compaction and subsequent vesiculation of myelin sheaths coupled with a moderate influx of lipid laden macrophages is consistent with pathological findings of primary demyelination. Importantly, neurofilament integrity and organization of the axoplasm appeared normal; staining for MBP and neurofilament proteins in late post radiation rat and human samples confirmed a loss of myelin with apparent preservation of axons.

In summary this study demonstrates permanent suppression of the SVZ stem cell compartment following radiation as well as an early and sustained loss of oligodendrocyte precursor cells with subsequent delayed demyelination. The detailed analysis of various cell populations over time reveals potential therapeutic windows that could target the recovery phases of neural precursors post injury prior to the occurrence of structural damage to the myelin sheaths. The rat model is validated by similar findings in human tissue. Based on this model, therapeutic strategies may be directed at reducing initial precursor cell loss or possibly at replacing the lost precursor cells via transplantation of primary or stem cell derived oligodendrocyte precursors as a means of preventing late radiation-induced demyelination.

## Supporting Information

Figure S1 Immunohistochemistry of coronal sections through the SVZ at various times post radiation (A). Quantitative measurements shown in (B) demonstrate significant suppression on day 1 that is maintained well below normal controls with a minor recovery peak at 9 months post radiation, also illustrated in (A). BrdU/NG2 kinetics in the corpus callosum (CC) and cortex (Cx) are noted for a more sustained recovery of cell numbers to approach those of normal age-matched controls, particularly beyond 9 months post XRT. (*** p<0.001; ** p<0.01; * p<0.05; ANOVA). Bars = SEM. Scale bar in (A) corresponds to 50 µm in all panels except 12 months where it corresponds to 100 µm.
(3.14 MB TIF)

Figure S2 Rat samples in (A) demonstrate progressive loss of O1 noted at 2 months post XRT with further decrease and no recovery at 15 months post radiation. MAG, a marker associated with more mature oligodendrocytes, is depleted only at late time points. Human white matter samples in (B) were acquired from non-irradiated (normal brain) and irradiated specimens up to 7 months post XRT (labeled “early”) and between 9 months and 7 years (labeled “late”). Immunohistochemistry for markers of intermediate/late oligodendrocyte progenitors O1, Galc and MAG show a similar pattern of delayed loss of expression with profound loss and no evidence of recovery in the late phases. Scale bar corresponds to 100 µm in all panels.
(6.67 MB TIF)

Figure S3 Panels of human (A) and rat (B) control and irradiated tissues at 14 months post XRT in both specimens. Immunohistology for MBP demonstrates loss of myelin (red) without obvious loss of neurofilament (green). Antibodies against NF-70 were used for human tissues and NF-M for rat tissues. Scale bar corresponds to 100 µm in all panels. Representative sections at the level of the hippocampal commissure and dorsal fornix in the rat are shown in the normal age-matched and irradiated rat brain in (A) and (B) respectively. There is severe focal necrosis with myelin (red) and cell loss (DAPI, blue nuclei). Two of the human specimens were acquired in the context of symptomatic radiation necrosis. Histological assessment (H&E) demonstrates pale-staining foci of necrosis without surrounding hypercellularity (C) and amorphous necrotic debris with scattered macrophages in (D). Scale bars correspond to 100 µm in (A), (B) and (C) and to 50 µm in (D).
(6.13 MB TIF)

Figure S4 Necrosis is seen in some rat tissues beyond 15 months and in select patients presenting with clinical symptoms post radiation. Representative sections at the level of the hippocampal commissure and dorsal fornix in the rat are shown in the normal age-matched and irradiated rat brain in (A) and (B) respectively. There is severe focal necrosis with myelin (red) and cell loss (DAPI, blue nuclei). Two of the human specimens were acquired in the context of symptomatic radiation necrosis. Histological assessment (H&E) demonstrates pale-staining foci of necrosis without surrounding hypercellularity (C) and amorphous necrotic debris with scattered macrophages in (D). Scale bars correspond to 100 µm in (A), (B) and (C) and to 50 µm in (D).
(5.01 MB TIF)
