# Supplementary figures and images for: Long-Term Impact of Radiation on the Stem Cell and Oligodendrocyte Precursors in the Brain
Source: PLoS One. 2007 Jul 11;2(7):e588. doi: 10.1371/journal.pone.0000588 (PMC1913551; doi:10.1371/journal.pone.0000588)

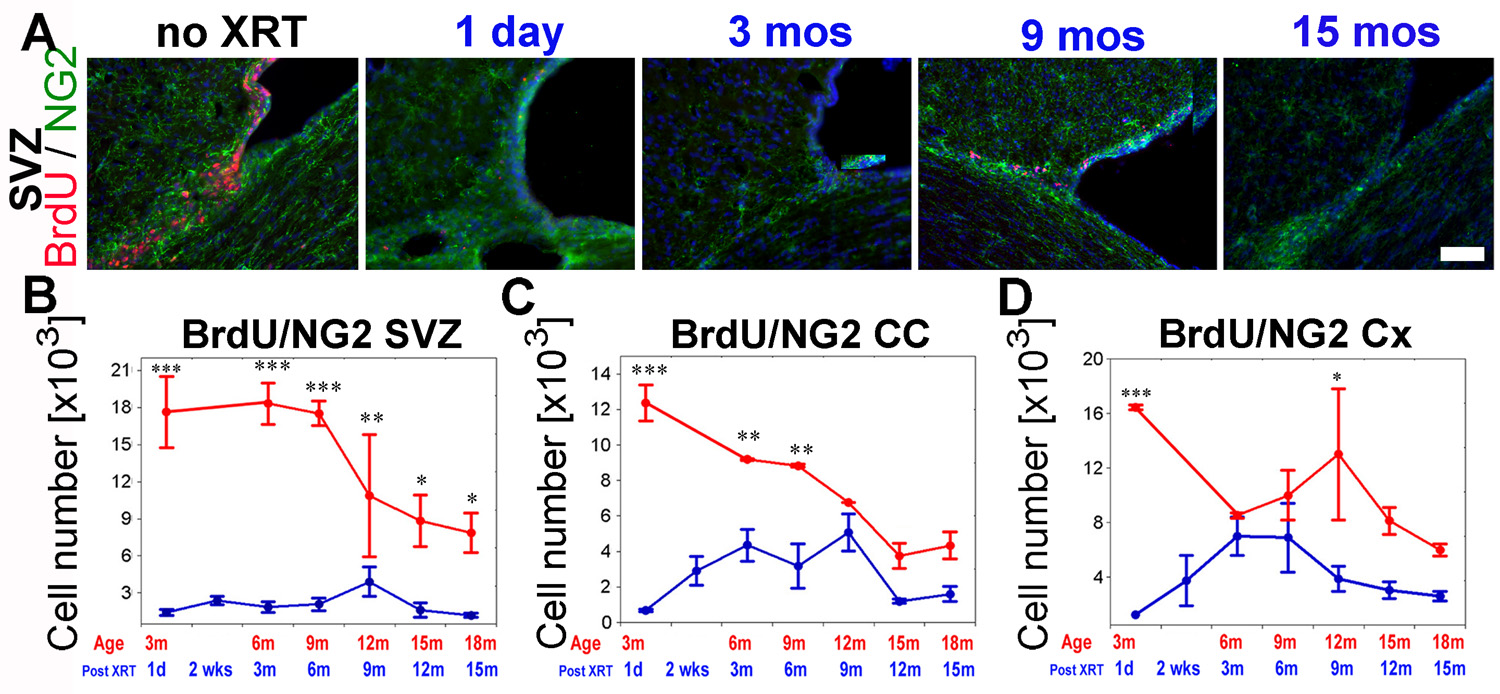

Supplement: Figure S1 — Immunohistochemistry of coronal sections through the SVZ at various times post radiation (A). Quantitative measurements shown in (B) demonstrate significant suppression on day 1 that is maintained well below normal controls with a minor recovery peak at 9 months post radiation, also illustrated in (A). BrdU/NG2 kinetics in the corpus callosum (CC) and cortex (Cx) are noted for a more sustained recovery of cell numbers to approach those of normal age-matched controls, particularly beyond 9 months post XRT. (*** p<0.001; ** p<0.01; * p<0.05; ANOVA). Bars = SEM. Scale bar in (A) corresponds to 50 µm in all panels except 12 months where it corresponds to 100 µm. (3.14 MB TIF) [file pone.0000588.s001.tif]

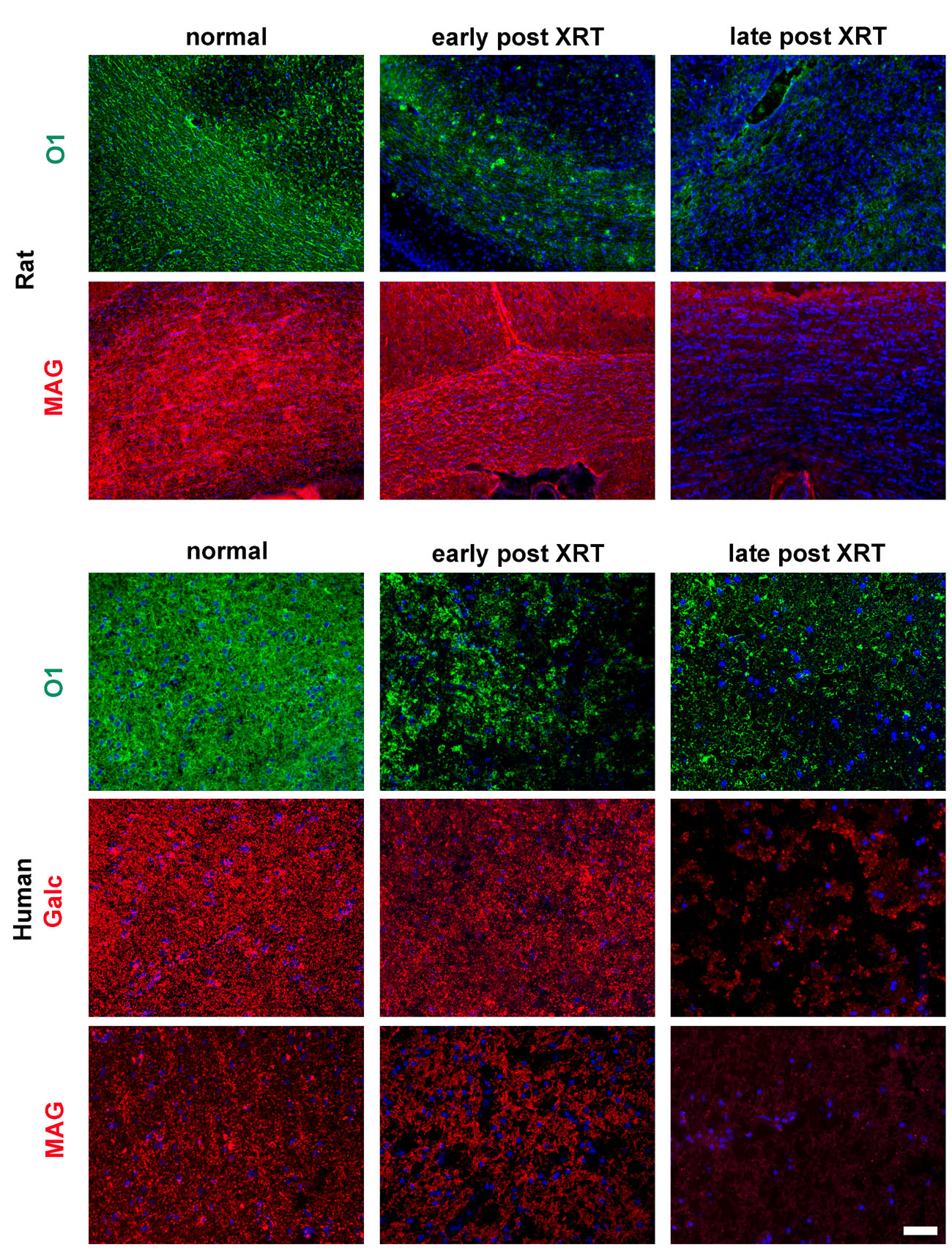

Supplement: Figure S2 — Rat samples in (A) demonstrate progressive loss of O1 noted at 2 months post XRT with further decrease and no recovery at 15 months post radiation. MAG, a marker associated with more mature oligodendrocytes, is depleted only at late time points. Human white matter samples in (B) were acquired from non-irradiated (normal brain) and irradiated specimens up to 7 months post XRT (labeled “early”) and between 9 months and 7 years (labeled “late”). Immunohistochemistry for markers of intermediate/late oligodendrocyte progenitors O1, Galc and MAG show a similar pattern of delayed loss of expression with profound loss and no evidence of recovery in the late phases. Scale bar corresponds to 100 µm in all panels. (6.67 MB TIF) [file pone.0000588.s002.tif]

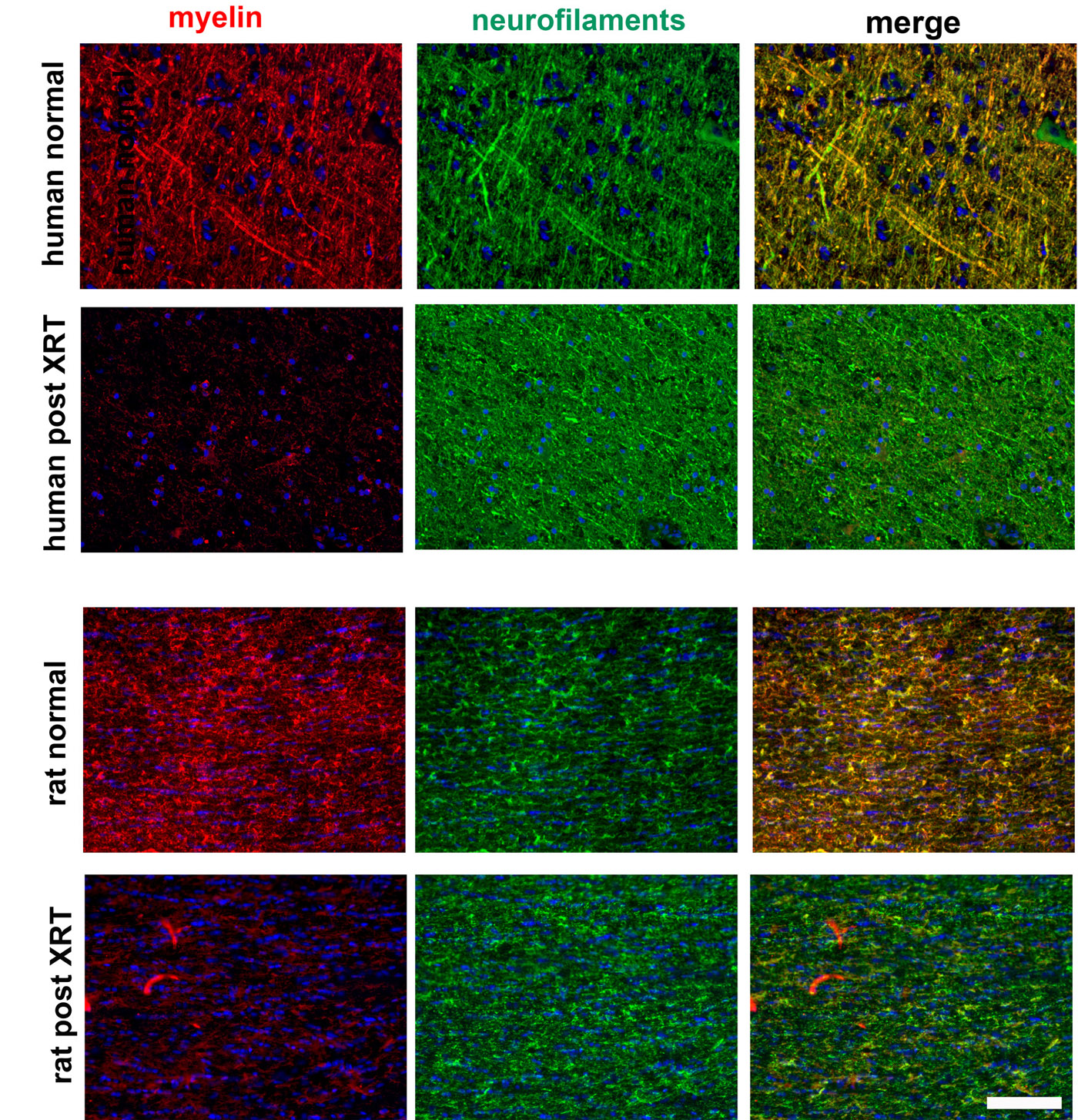

Supplement: Figure S3 — Panels of human (A) and rat (B) control and irradiated tissues at 14 months post XRT in both specimens. Immunohistology for MBP demonstrates loss of myelin (red) without obvious loss of neurofilament (green). Antibodies against NF-70 were used for human tissues and NF-M for rat tissues. Scale bar corresponds to 100 µm in all panels. Representative sections at the level of the hippocampal commissure and dorsal fornix in the rat are shown in the normal age-matched and irradiated rat brain in (A) and (B) respectively. There is severe focal necrosis with myelin (red) and cell loss (DAPI, blue nuclei). Two of the human specimens were acquired in the context of symptomatic radiation necrosis. Histological assessment (H&E) demonstrates pale-staining foci of necrosis without surrounding hypercellularity (C) and amorphous necrotic debris with scattered macrophages in (D). Scale bars correspond to 100 µm in (A), (B) and (C) and to 50 µm in (D). (6.13 MB TIF) [file pone.0000588.s003.tif]

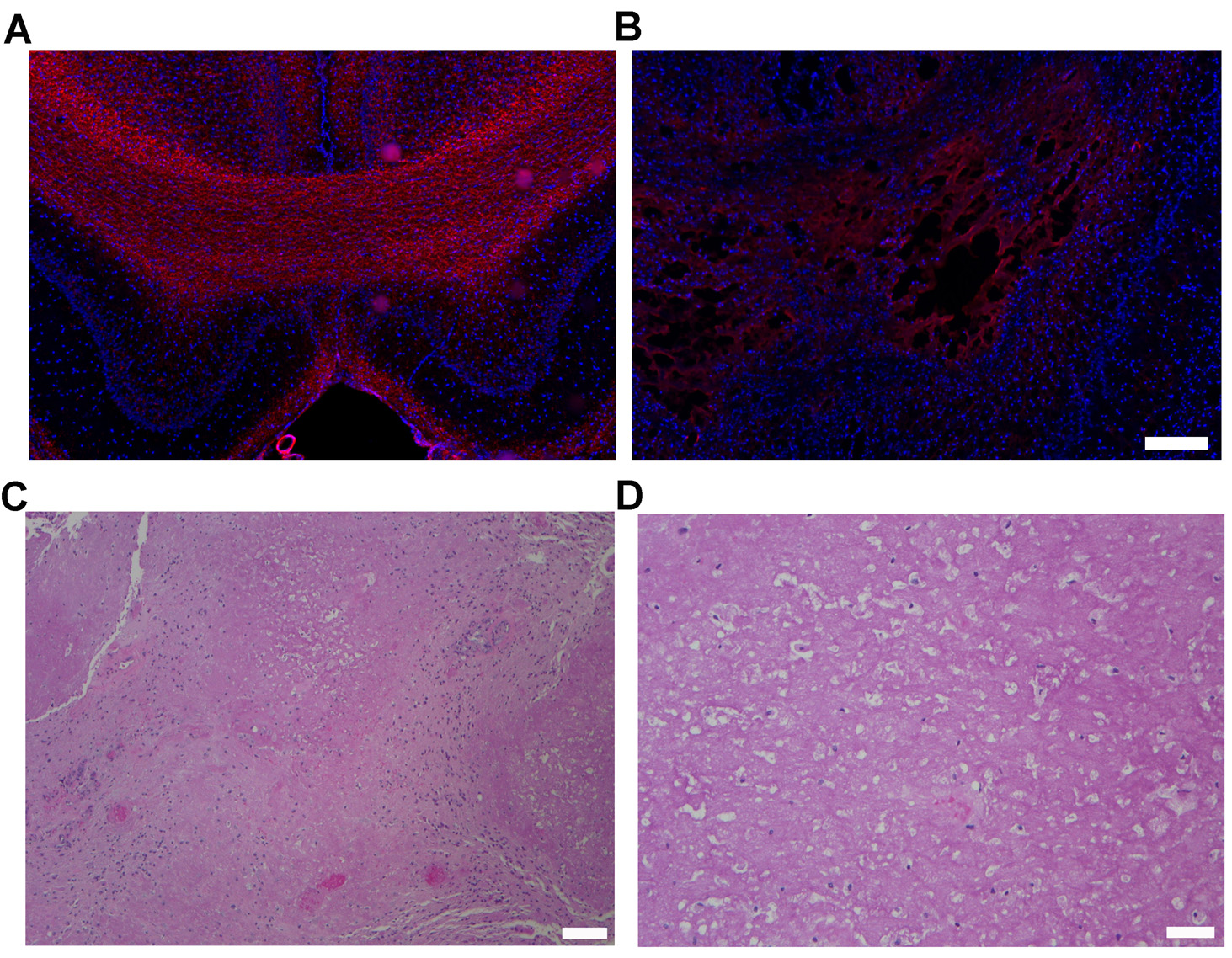

Supplement: Figure S4 — Necrosis is seen in some rat tissues beyond 15 months and in select patients presenting with clinical symptoms post radiation. Representative sections at the level of the hippocampal commissure and dorsal fornix in the rat are shown in the normal age-matched and irradiated rat brain in (A) and (B) respectively. There is severe focal necrosis with myelin (red) and cell loss (DAPI, blue nuclei). Two of the human specimens were acquired in the context of symptomatic radiation necrosis. Histological assessment (H&E) demonstrates pale-staining foci of necrosis without surrounding hypercellularity (C) and amorphous necrotic debris with scattered macrophages in (D). Scale bars correspond to 100 µm in (A), (B) and (C) and to 50 µm in (D). (5.01 MB TIF) [file pone.0000588.s004.tif]
